# Expression of Concern: Deep CNN-based detection of cardiac rhythm disorders using PPG signals from wearable devices

**DOI:** 10.1371/journal.pone.0354777

**Published:** 2026-07-28

**Authors:** The PLOS One Editors

After this article [1] was published, concerns were noted that the articles cited as References 11 and 35 were retracted after [1] was submitted and before [1] was published.

In addition, the *PLOS One* Editors have concerns about the article’s compliance with the PLOS Authorship policy.

In light of the cumulative issues, the *PLOS One* Editors issue this Expression of Concern. Readers are advised to interpret the article [1] with caution.

## References

[1] Bulut MG, Unal S, Hammad M, Pławiak P. Deep CNN-based detection of cardiac rhythm disorders using PPG signals from wearable devices. PLoS One. 2025;20(2):e0314154. doi: 10.1371/journal.pone.0314154 39937744 PMC11819536

