# Supplementary material for: Deep CNN-based detection of cardiac rhythm disorders using PPG signals from wearable devices
Source: PLoS One. 2025 Feb 12;20(2):e0314154. doi: 10.1371/journal.pone.0314154 (PMC11819536; doi:10.1371/journal.pone.0314154)
Supplement: Attachment — Submitted filename: R2.pdf [file pone.0314154.s002.pdf]

Abstract

1) In the added lines 41-44, "Datasets are available for download on" is repeated twice. Further, the entire sentence is linked, not only the specific links to the repositories.

Introduction

2) The paragraph (lines 64-73) about the difference between 2-leads ECG and 12-leads ECG was inserted in the introduction that is hard to follow now. This paragraph is useful to understand the results of the study, thus authors should move it to the discussion section.

PPG Data Set

3) Can authors add the measurement units on the y-axis label in figure 2?

Discussion

4) Even if future works aim to train and validate their 1D CNN model on a more balanced population in terms of sex and age, the authors should here discuss this crucial aspect regarding the clinical applicability of their work. As commented int the first revision, the population is sex-unbalanced (28 male vs 9 female). The incidence of atrial fibrillation is greater in males than in females, but this gap closes with advancing age (population in the present study aged between 51 and 90). Further sex-related differences in photoplethysmography signals exist.
   At the present form, the proposed method is limited and it is not clinically applicable to a general population.
   Authors should deeply discuss these limitations in the discussion section.

5) In lines 552-553, authors stated "Signal quality can be affected by motion artifacts, electrode contact issues, and skin variability, making it difficult to obtain accurate PPG signals". PPG is not acquired by electrodes, can authors correct their statement?

6) What do authors refer to with "invasive electrode placement (For Invasive EKG Devices)"?

   Non-invasive Electrocardiographic monitoring can be performed by dry electrodes and capacitive sensors for ECG, which can be incorporated in wearable and portable devices. The authors stated that ECG acquisition require contact with the subject, but the PPG acquisition on the chest or on the wrist through smartwatches also require contact with the device and possibly discomfort.

   Can Authors provide a more realistic and balanced comparison of wearable devices for ECG and PPG acquisition systems?

7) In table 4, authors should add ECG segment to the feature set of their work.
