# Supplementary material for: Deep CNN-based detection of cardiac rhythm disorders using PPG signals from wearable devices
Source: PLoS One. 2025 Feb 12;20(2):e0314154. doi: 10.1371/journal.pone.0314154 (PMC11819536; doi:10.1371/journal.pone.0314154)
Supplement: Attachment — Submitted filename: Response to Reviewers.docx [file pone.0314154.s001.docx]

**Manuscript Ref. No:** PONE-D-24-02099

**Title of Paper	:** Deep CNN model for AF and PAC detection with PPG signals from Wearable Devices

**Authors:** Miray Gunay Bulut, Sencer Unal, Mohamed Hammad, Paweł Pławiak

First of all, we would like to thank the reviewers and the editorial board for their valuable remarks and constructive comments on the manuscript. Remarks and comments suggested by reviewers have been applied in the manuscript and responses to reviewers are also given in detail. The revised form of the manuscript is uploaded through the journal web page.

We have made a substantial revision of our paper (the revised parts are marked in red), and provided detailed and itemized responses to the comments of reviewers.

**AUTHOR’S RESPONSE TO COMMENTS OF THE REVIEWER#1**

The paper presents promising findings; however, it requires major revision in the following areas:

• The structure of the paper is confusing. In the introduction, sections are used to discuss literature, which should instead be part of the related work. A similar issue occurs in the Discussion, where the authors review literature instead of solely comparing results.

We would like to thank to the reviewer for this valuable contribution. Based on the comments made, we added more detailed information about the results to the *“Discussion”* section.

• The language used in the paper, particularly in the introduction, is unclear and needs rephrasing and better use of conjunctions.

Based on the comments, the article was reviewed and revised.

• It is not clear how the 2-channel ECG were used as an input.

Based on the comments, we added additional information about ECG to the *“PPG data set section”.*

• Additional points of ambiguity will be listed in the comments below.

**1-Comment of Reviewer1:** 1. Line 51: Traditionally, electrocardiogram (ECG) [3]

Comment: The placement of the reference should be moved to later in the sentence.

**Author’s Response**

We would like to thank to the reviewer for this valuable contribution. According to this comment, we corrected the sentence in the “*Introduction*” section as follows.

*“…Traditionally, electrocardiogram (ECG) signals are used to detect arrhythmias [3].”*

**2-Comment of Reviewer1:** 2. Line 64: These signals are typically collected by shining a light source (usually an LED)

Comment: LED is an abbreviation that should be defined before used, and it is better to list the wavelength used.

**Author’s Response**

We would like to the reviewer for this contribution. According to this comment we added following sentences to “*Introduction*” section.

*“…These signals are typically collected by shining a light source (usually a Light Emitting Diode (LED) with a wavelength range of 600-1000 nm) onto a part of the body (usually a fingertip or earlobe) and measuring the amount of light absorbed by the underlying tissue.”*

**3-Comment of Reviewer1:** 3. Line 79: Deep learning has emerged as a powerful tool in the field of medical signal processing, particularly suitable for arrhythmia detection due to its ability to learn complex patterns and reduce the need for manual feature engineering.

Comment: Deep learning based on Convolutional neural networks does not require manual features; however deep learning can depend on manual features.

**Author’s Response**

We would like to the reviewer for this contribution. According to this comment, we corrected the sentence in the “*Introduction*” section as follows.

*“...Deep learning has emerged as a powerful tool in the field of medical signal processing, particularly suitable for arrhythmia detection due to its ability to learn complex patterns. While deep learning based on Convolutional Neural Networks (CNNs) does not require manual feature engineering, other deep learning approaches can still depend on manual features.”*

**4-Comment of Reviewer1:** 4. Line 118: The same model is also applied to the ECG channels within the dataset, Comment: when the authors say the same model, do they mean the same architecture or after performing fine tuning?

**Author’s Response**

We would like to thank to the reviewer for this valuable contribution. According to this comment, we corrected the sentence in the “*Introduction*” section as follows.

*“…The same 17-layer 1D-CNN architecture applied to PPG signals is also applied to the ECG channels within the dataset without any modifications, and the results are compared.”*

**5-Comment of Reviewer1:** 5. Line 132: The integration of PPG signals and the non-invasive nature of the approach:

Comment: PPG is a non-invasive signal what do the author mean by this sentence?

**Author’s Response**

We would like to thank to the reviewer for this valuable contribution. According to this comment we added following sentences to “*Introduction*” section.

*“…The integration of PPG signals, which involve sensors that do not penetrate the skin, contributes to improved patient comfort by addressing concerns related to electrode placement on the skin and promoting a more patient-friendly monitoring experience.”*

**6-Comment of Reviewer1:** 6. Line 146: In the literature, various studies have employed deep learning approaches for disease detection using PPG signals. For example, Chiang et al. [10], used a support vector machine (SVM) classifier to evaluate the quality of arteriovenous fistulas in haemodialysis patients using a PPG sensor device. In another study, Uçar et al. [35], proposed a new sleep staging method based on the k-nearest neighbour (KNN) classification algorithm using PPG signals. Kwon et al.

Comment: SVM and KNN are not deep learning methods, but machine learning ones.

**Author’s Response**

We would like to thank to the reviewer for this valuable contribution. According to this comment, we corrected the sentence in the “*Related Works*” section as follows.

*“…In the literature, machine learning and deep learning approaches have been employed for disease detection using PPG signals in various studies.”*

**7-Comment of Reviewer1:** 7. Line 222: Fig 1. Representation of ECG signals in the dataset: a) AF, b) NSR, and c) PAC with representation of PPG signals in the dataset: d) AF e) NSR, and f) PAC.

Comment: needs to be rephrased.

**Author’s Response**

We would like to thank to the reviewer for this valuable contribution. According to this comment, we corrected the sentence in the “*PPG data set*” section as follows.

*“…Fig 1. Representations of a) NSR, b) AF, and c) PAC signals in the ECG dataset, along with d) NSR, e) AF, and f) PAC signals in the PPG dataset.”*

**8-Comment of Reviewer1:** 8. Line 240: features from the input signal, al-lowing the model.

Comment: A typo in “al-lowing”.

**Author’s Response**

We would like to thank to the reviewer for this valuable contribution. According to this comment, we corrected the sentence in the “*Proposed 1D-CNN model*” section as follows.

*“…Each layer in the CNN extracts increasingly complex features from the input signal, allowing the model to learn hierarchical representations that are essential for effective signal classification.”*

**9-Comment of Reviewer1:** 9. Line 242: CNN employs parameters such as filters, pooling layers, and activation functions to enhance its ability to recognize distinctive patterns in the PPG data. Through the training process, the model optimizes these parameters to minimize classification errors.

Comment: filters, pooling layers, and activation functions are not parameters that optimized through the training process as these parameters do not change its value after the training initialized.

**Author’s Response**

We would like to thank to the reviewer for this valuable contribution. According to this comment, we corrected the sentence in the “*Proposed 1D-CNN model*” section as follows.

*“…Furthermore, the 1D CNN employs elements such as filters, pooling layers, and activation functions to enhance its ability to recognize distinctive patterns in the PPG data. Through the training process, the model optimizes the weights and biases to minimize classification errors and increase overall performance.”*

**10-Comment of Reviewer1:** In Figure 2 numbers of Kernal size and numbers of filters are not easy to interpret, maybe the authors find another way to express it.

**Author’s Response**

We would like to thank to the reviewer for this valuable contribution. According to this comments we edited Figure 2 as follows in the “*Proposed 1D-CNN model*” section.

*“…*

**11-Comment of Reviewer1:** In Table 1: have two separated columns for kernel size and unit and rename the unit to number of filters.

**Author’s Response**

We would like to thank to the reviewer for this valuable contribution. According to this comments we edited Table 1 as follows in the “*Proposed 1D-CNN model*” section.

*“*

| **Layer** | **Layer Name** | **Kernel Size** | **Number of Filters** | **Other Layer Parameters** |
| --- | --- | --- | --- | --- |
| 1 | Conv1D | 50 | 128 | Activation = ReLU, Strides = 1 |
| 2 | MaxPooling1D | - | - | Pooling Size = 1, Strides = 2 |
| 3 | Conv1D | 20 | 64 | Activation = ReLU, Strides = 1 |
| 4 | MaxPooling1D | - | - | Pooling Size = 1, Strides = 2 |
| 5 | Conv1D | 20 | 64 | Activation = ReLU, Strides = 1 |
| 6 | MaxPooling1D | - | - | Pooling Size = 1, Strides = 2 |
| 7 | Conv1D | 5 | 128 | Activation = ReLU, Strides = 1 |
| 8 | Conv1D | 5 | 128 | Activation = ReLU, Strides = 1 |
| 9 | MaxPooling1D | - | - | Pooling Size = 1, Strides = 2 |
| 10 | Conv1D | 3 | 256 | Activation = ReLU, Strides = 1 |
| 11 | Conv1D | 3 | 256 | Activation = ReLU, Strides = 1 |
| 12 | MaxPooling1D | - | - | Pooling Size = 1, Strides = 2 |
| 13 | Conv1D | 3 | 512 | Activation = ReLU, Strides = 1 |
| 14 | MaxPooling1D | - | - | Pooling Size = 1, Strides = 2 |
| 15 | Flatten | - | - | - |
| 16 | Dense | - | 512 | ReLU |
| 17 | SoftMax | - | - | Softmax |

**12-Comment of Reviewer1:** 12. Line 261: including label encoding, one-hot encoding,

Comment: are these two the same thing? Then why it is mentioned twice?

**Author’s Response**

We would like to thank to the reviewer for this valuable contribution.

Label Encoding: Converts categorical data into numerical values. For example, it transforms categories like "red," "green," and "blue" into numerical values such as 1, 2, and 3, respectively.

One-Hot Encoding: Converts each category into binary vectors. Each category is represented by a vector with a 1 in the position corresponding to that category and 0s elsewhere. For example, the categories "red," "green," and "blue" can be encoded as follows:

"red" -> [1, 0, 0]

"green" -> [0, 1, 0]

"blue" -> [0, 0, 1]

These two methods offer different advantages and disadvantages for various situations. Therefore, label encoding and one-hot encoding are not the same and are distinct encoding methods.

According to this comment we added following sentences to “*Experimental Setup*” section.

*“…Preprocessing steps were applied to the PPG and ECG signals in the dataset, including min-max normalization and label encoding.”*

**13-Comment of Reviewer1:** 13. Line 261: feature scaling (min-max normalization)

Comment: do the authors mean scaling the signal?

**Author’s Response**

We would like to thank to the reviewer for this valuable contribution.

*In our code, the line “scaler = MinMaxScaler(feature_range=(0, 1))” scales the signal. This line performs an operation that transforms data features into a specific range (typically [0, 1]). In other words, it rescales the values of data points according to a predefined min-max range.*

**14-Comment of Reviewer1:** 14. Line 269: The 1D CNN model was trained on labelled datasets consisting of annotated ECG and PPG records with corresponding arrhythmia types.

Comment: why mention the data here again where the paragraph was discussing the data earlier?

**Author’s Response**

We would like to thank to the reviewer for this valuable contribution. We removed this sentence from the “*Experimental Setup*” section:*“The 1D CNN model was trained on labeled datasets consisting of annotated ECG and PPG records with corresponding arrhythmia types”*

**15-Comment of Reviewer1:** 15. Line 274: Sensitivity, also known as recall, measures the model's ability to correctly identify positive samples among all true positive examples.

Comment: The definition needs to be rephrased as this is not accurate.

**Author’s Response**

We would like to thank to the reviewer for this valuable contribution. According to this comment we added following sentences to “*Experimental Setup*” section.

*“…Sensitivity refers to the proportion of correctly predicted observations in a class among all observations belonging to that class. In other words, it measures the classifier's ability to correctly identify data that is truly positive.”*

**16-Comment of Reviewer1:** 16. Line 352: The confusion matrix shows the number of true positives, false positives, true negatives, and false negatives for each class:

Comment: Why defining the confusion matrix here where it was introduced earlier in line 328?

**Author’s Response**

We would like to thank to the reviewer for this valuable contribution. According to this comment we removed this sentence from the *“Classification Results”* section: *“The confusion matrix shows the number of true positives, false positives, true negatives, and false negatives for each class.”*

**17-Comment of Reviewer1:** 17. Line 376: In this study, a PPG dataset prepared by the University of Massachusetts Medical Center (UMMC) was utilized. Table 4 provides a summary of studies focusing on the analysis of PPG signals. Lemay et al. [27],

Comment: the moving to the table of summary is weird and require rephrasing.

**Author’s Response**

We would like to thank to the reviewer for this valuable contribution. According to this comment, we corrected the sentence in the “*Discussion*” section as follows.

*“…Table 4 provides a summary of a comparison of studies developed for the detection of cardiovascular diseases from signals obtained from wearable devices with machine learning methods.”*

**18-Comment of Reviewer1:** 18. Line 395: achieved an impressive accuracy rate of 95.17% for PPG signals.

Comment: It is not appropriate to call your results impressive.

**Author’s Response**

We would like to thank to the reviewer for this valuable contribution. According to this comment, we changed and rewrote the following sentence in the *“Discussion”* section.

*“…The developed 17-layer 1D CNN model provided an accuracy rate of 95.17% for PPG signals.”*

**19-Comment of Reviewer1:** 19. Line 398: single-hot encoding was performed.

Comment: Do the authors mean “One-hot” why replacing One by single?

**Author’s Response**

According to this comment, we changed and rewrote the following sentence in the *“Discussion*” section.

*“…Only preprocessing steps such as min-max normalization and label encoding were performed on the PPG and ECG signals.”*

**20-Comment of Reviewer1:** 20. Line 402: The advantages of this study can be listed as follows:

Comment: The advantages keep mentioned early, although the dataset does not specify how early in stages were the patients at, also listing “Limited and imbalanced data distribution” as an advantage is not understandable.

**Author’s Response**

We would like to thank to the reviewer for this valuable contribution. "Limited and imbalanced data distribution" is given in the disadvantages section of the *“Discussion”* section. That's why it wasn't changed.

*“The disadvantages of this study are as follows:*

- *Limited and imbalanced data distribution (insufficiency of data in the PAC class).”*

**Manuscript Ref. No:** PONE-D-24-02099

**Title of Paper	:** Deep CNN model for AF and PAC detection with PPG signals from Wearable Devices

**Authors:** Miray Gunay Bulut, Sencer Unal, Mohamed Hammad, Paweł Pławiak

First of all, we would like to thank the reviewers and the editorial board for their valuable remarks and constructive comments on the manuscript. Remarks and comments suggested by reviewers have been applied in the manuscript and responses to reviewers are also given in detail. The revised form of the manuscript is uploaded through the journal web page.

We have made a substantial revision of our paper (the revised parts are marked in red), and provided detailed and itemized responses to the comments of reviewers.

**AUTHOR’S RESPONSE TO COMMENTS OF THE REVIEWER#2**

**1-Comment of Reviewer2:** The sentence ‘PPG signals enable recording and continuous monitoring over extended periods, providing access to heart signals at any time’ at line 73-75 is not clear. Can Authors reformulate it?

**Author’s Response**

We would like to thank to the reviewer for this valuable contribution. According to this comment, we corrected the sentence in the “*Introduction*” section as follows.

*“…Smartwatches enable the recording and continuous monitoring of PPG signals over extended periods.”*

**2-Comment of Reviewer2:** The abbreviation NSR was note introduced before its use. Can Authors introduce it where they write normal sinus rhythm for the first time?

**Author’s Response**

We would like to thank to the reviewer for this valuable contribution. According to this comment we added following sentence to “*Introduction*” section.

*“…normal sinus rhythm (NSR)”*

**3-Comment of Reviewer2:** The population is sex-unbalanced (28 male vs 9 female). The incidence of atrial fibrillation is greater in males than in females, but this gap closes with advancing age (population in the present study aged between 51 and 90). Further sex-related differences in photoplethysmography signals exist. I suggest to train and validate the proposed 1D CNN on a sex-balanced population. At least, Authors should deeply discuss this limitation in the discussion.

**Author’s Response**

We would like to thank our referee for his valuable contributions. In our future work, our proposed 1D CNN model will be trained and validated on a more balanced population in terms of gender and age.

**4-Comment of Reviewer2:** The acquisition protocol is not clear. Are the subjects in a laboratory or in a daily life situation? How much did the recordings last? Can Authors add the description of setting and timing of acquisition?

**Author’s Response**

We would like to thank to the reviewer for this valuable contribution. According to this comment we added following sentences to “*PPG Data Set*” section.

*“…Patients with arrhythmia registered at the University of Massachusetts Medical Center wore a Gear S3 smartwatch and an ECG probe while being allowed to perform their normal activities for 14 consecutive days/24 hours. The data were obtained by charging the smartwatches daily and replacing the ECG device's battery on the 7th day.”*

**5-Comment of Reviewer2:** Are subjects undergoing any cardiac therapy?

**Author’s Response**

We would like to thank to the reviewer for this valuable contribution. Subjects are arrhythmia patients registered with the University of Massachusetts Medical Center.

**6-Comment of Reviewer2:** In figure 1, are the PPG windows corresponding to the ECG windows? In the PPG each pulse corresponds to a cardiac beat, thus if there are 2 cardiac beats in the ECG I expect two pulses in the PPG window. Can Authors check and correct the figure?

**Author’s Response**

We would like to thank to the reviewer for this valuable contribution. According to this comments we edited Figure 1 as follows in the “*PPG Data Set*” section.

**7-Comment of Reviewer2:** In line 240 ‘al-lowing’ should be allowing.

**Author’s Response**

We would like to thank to the reviewer for this valuable contribution. According to this comment, we corrected the sentence in the “*Proposed 1D-CNN Model*” section as follows.

*“…Each layer in the CNN extracts increasingly complex features from the input signal, allowing the model to learn hierarchical representations that are essential for effective signal classification.”*

**8-Comment of Reviewer2:** This section contains methodological information, I suggest to move it in the method chapter.

**Author’s Response**

We would like to thank to the reviewer for this valuable contribution. According to this comment, we moved the following section from Experimental result chapter to the Materials and Methods chapter.

*“…Preprocessing steps were applied to the PPG and ECG signals in the dataset, in-cluding min-max normalization and label encoding. All signals were divided into 30-second lengths without overlap. The ECG signals, filtered with a Bandpass filter, and the PPG signals, which were in raw form, were used, as obtained from the data in [38].”*

**9-Comment of Reviewer2:** In the list of metrics, line 272, both sensitivity and recall appear. Authors should select only one metric name and use it consistently along the paper.

**Author’s Response**

We would like to thank to the reviewer for this valuable contribution. Sensitivity and recall are formulaically the same concepts, but they are used in different contexts. Both measure the ability of a model or test to accurately identify positive examples. They were used in different contexts in our study. That's why they are expressed separately in our study.

**10-Comment of Reviewer2:** The two ECG channels contains information of the same cardiac electrical activity in corresponding windows but they provide different results. May it depend on electrode position? Can Authors discuss this result in detail and elucidate on the different electrode position and ECG morphology in the two channels?

**Author’s Response**

We would like to thank to the reviewer for this valuable contribution. According to this comment we added following sentences to “*Introduction*” section.

*“…Electrodes in standard 12-lead ECGs are placed on the limbs (right arm, left arm, right leg, left leg) and the chest (V1-V6). Each electrode placement captures electrical signals from various angles, providing a comprehensive view of the heart's activity. The morphology of ECG waves (P wave, QRS complex, and T wave) varies depending on the electrode position, reflecting different aspects of the heart's electrical function. The 2-lead ECG used in this study is a simple type of electrocardiogram (ECG) that uses two electrodes to record the heart's electrical activity. This type of ECG is typically used for more basic and quick assessments. In 2-lead ECGs, electrodes are generally placed on the chest or limbs. It provides less information compared to a 12-lead ECG.”*

According to this comment we added following sentences to “*Classification Results*” section.

*“...The idea that different classification results from signals coming from dif-ferent ECG channels may be due to electrode position. Each lead captures the heart's electrical activity from a specific viewpoint, resulting in different ECG morphologies. For example, while one lead captures electrical activity from a particular part of the heart, another lead collects data from a completely different perspective. This diversity leads to each lead having different signal characteristics.*

*Changes in electrode placement cause these signal characteristics to vary, which can affect the outcomes of automatic classification algorithms. When algorithms evaluate these different signal characteristics, they use the unique perspec-tive provided by each lead for classification. Therefore, the different classification results obtained from two ECG channels stem from the differences in electrode positions and the unique perspectives they provide on the heart's electrical activity.*

*In summary, the positioning of electrodes can directly influence the morphology of ECG signals and, consequently, the results of classification algorithms. This highlights the critical importance of electrode placement for accurate and consistent ECG analyses.*

**11-Comment of Reviewer2:** In the discussion section Authors should discuss more in deep their results and limitations. Further Authors should provide a more comprehensive comparison with literature.

**Author’s Response**

We would like to thank to the reviewer for this valuable contribution. According to this comment we added following sentences to “*Discussion*” section.

*“…In addition, different classification results were obtained from the signals received from both channels of the ECG. The reason is that the signals received from both channels are different. The electrode positions of the two channels are different. Different electrode position leads to different ECG morphology. Therefore, different signals are obtained from different channels. In this way, different signals were obtained in our study and different classification results were obtained and the results were compared.”*

**12-Comment of Reviewer2:** Can Authors discuss the adaptability of the presented CNN to PPG signals acquired in different body locations (ex. Finger, wrist, ear..)?

**Author’s Response**

We would like to thank to the reviewer for this valuable contribution. According to this comment we added following sentences to “*Discussion*” section.

*“…In this study, we performed arrhythmia detection using a CNN model applied to PPG signals obtained from the chest and wrist regions. Our results indicate that our designed model can successfully detect arrhythmia using data from these regions. However, it is important to evaluate the adaptability of PPG signals from different body regions to our model for the future expansion and application of our study. Studies focusing on the impact of PPG signals from different areas such as the finger and ear on model performance could enhance its generalization ability and expand its potential for clinical application. This is because PPG signals from different body regions can vary due to physiological and anatomical differences such as tissue density, blood flow, and environmental factors. Regions like the finger and ear may present significant differences in signal quality and patterns.*

*The findings from our study suggest avenues for further research into how obtaining PPG signals from different body regions may influence model performance. Such advanced studies could improve the reliability of PPG-based arrhythmia detection methods and broaden their utility in clinical applications. In our future work, assessing the adaptability of PPG signals from different body regions to our CNN model holds potential for enhancing arrhythmia detection performance. This will help us better understand the effectiveness and generalizability of these methods in clinical practice.”*

**13-Comment of Reviewer2:** Authors continuously compare the ECG and the PPG. Especially in lines 429 – 432 there is a list of ECG disadvantages “the use of ECG signals comes with disadvantages such as complexity and hardware requirements, contact and discomfort, limited long-term monitoring, invasive electrode placement, sensitivity to artifacts, and localization difficulties.” Although, the superficial ECG is non-invasive and the discomfort of wet electrodes has been overcome by dry electrodes and capacitive sensors for ECG, which are incorporated in wearable devices and everyday usable garments and objects. The PPG recording is also sensitive to artifacts. Can Authors provide a more realistic and balanced comparison of wearable devices for ECG and PPG acquisition systems?

**Author’s Response**

We would like to thank to the reviewer for this valuable contribution. According to this comment we made the necessary revisions in the “*Discussion*” section.
