# Supplementary material for: Deep CNN-based detection of cardiac rhythm disorders using PPG signals from wearable devices
Source: PLoS One. 2025 Feb 12;20(2):e0314154. doi: 10.1371/journal.pone.0314154 (PMC11819536; doi:10.1371/journal.pone.0314154)
Supplement: Attachment — Submitted filename: Response to Reviewers.docx [file pone.0314154.s003.docx]

**Manuscript Ref. No:** PONE-D-24-02099

**Title of Paper	:** Deep CNN model for AF and PAC detection with PPG signals from Wearable Devices

**Authors:** Miray Gunay Bulut, Sencer Unal, Mohamed Hammad, Paweł Pławiak

First of all, we would like to thank the reviewers and the editorial board for their valuable remarks and constructive comments on the manuscript. Remarks and comments suggested by reviewers have been applied in the manuscript and responses to reviewers are also given in detail. The revised form of the manuscript is uploaded through the journal web page.

We have made a substantial revision of our paper (the revised parts are marked in red), and provided detailed and itemized responses to the comments of reviewers.

**AUTHOR’S RESPONSE TO COMMENTS OF THE REVIEWER#1**

**1-Comment of Reviewer1:** 1. Typographical errors:

- Line 31: There is a typo in “mod-el”; it should be “model.”
- Line 221: “data set” should be written as “dataset.”

**Author’s Response**

We would like to thank to the reviewer for this valuable contribution. According to this comment, we corrected the sentences as follows.

*“In this study, a 1D-CNN* ***model*** *is proposed to detect arrhythmias using PPG signals.”*

*“PPG dataset”*

**2-Comment of Reviewer1:** Clarification needed on ECG leads:

In line 69, the authors state:

"The 2-lead ECG used in this study is a simple type of electrocardiogram (ECG) that uses two electrodes to record the heart's electrical activity."

While two electrodes can be used for a single-lead ECG (excluding the body reference electrode, however it was not excluded in the describtion of the 12-lead ecg), a two-lead ECG generally involves at least three electrodes. The dataset used by the authors does contain two channels, which does suggest that it is indeed a 2-lead ECG. This explanation, however, needs further clarification to avoid confusion. A correction is recommended.

**Author’s Response**

We would like to the reviewer for this contribution. According to this comment, we corrected the sentence in the “*Introduction*” section as follows.

*“The 2-lead ECG used in this study is a simple type of ECG that usually uses three electrodes to record the electrical activity of the heart. This type of ECG is used for more basic and quick assessments. In 2-lead ECGs, the electrodes are usually placed on the chest or limbs. It provides less information than a 12-lead ECG.”*

According to the suggestion of the Reviewer2, this paragraph has been moved to the discussion section.

**Manuscript Ref. No:** PONE-D-24-02099

**Title of Paper	:** Deep CNN model for AF and PAC detection with PPG signals from Wearable Devices

**Authors:** Miray Gunay Bulut, Sencer Unal, Mohamed Hammad, Paweł Pławiak

First of all, we would like to thank the reviewers and the editorial board for their valuable remarks and constructive comments on the manuscript. Remarks and comments suggested by reviewers have been applied in the manuscript and responses to reviewers are also given in detail. The revised form of the manuscript is uploaded through the journal web page.

We have made a substantial revision of our paper (the revised parts are marked in red), and provided detailed and itemized responses to the comments of reviewers.

**AUTHOR’S RESPONSE TO COMMENTS OF THE REVIEWER#2**

**1-Comment of Reviewer2:** 1) In the added lines 41-44, “Datasets are available for download on” is repeated twice. Further, the entire sentence is linked, not only the specific links to the repositories

**Author’s Response**

We would like to thank to the reviewer for this valuable contribution. According to this comment, we corrected the sentence in the “*Abstract*” section as follows.

*“…Datasets are available for download on https://www.synapse.org/pulsewatch."*

**2-Comment of Reviewer2:** The paragraph (lines 64-73) about the difference between 2-leads ECG and 12-leads ECG was inserted in the introduction that is hard to follow now. This paragraph is useful to understand the results of the study, thus authors should move it to the discussion section.

**Author’s Response**

We would like to thank to the reviewer for this valuable contribution. According to this comment, we moved this paragraph to the *“Discussion”* section.

*“In this study, different classification results were obtained from the signals obtained from both channels of the ECG. In particular, in standard 12-lead ECGs, the electrodes are placed on the limbs (right arm, left arm, right leg, left leg) and chest (V1-V6). Each electrode placement captures electrical signals from various angles, providing a comprehensive view of the heart's activity. The morphology of the ECG waves (P wave, QRS complex, and T wave) changes depending on the electrode position and reflects different aspects of the heart's electrical function. The 2-lead ECG used in this study is a simple type of ECG that usually uses three electrodes to record the electrical activity of the heart. This type of ECG is used for more basic and rapid assessments. In 2-lead ECGs, the electrodes are usually placed on the chest or limbs and provide less information than the 12-lead ECG. Considering these aspects, since the electrode positions of the two channels were different in our study, the signals obtained from the two channels were also different. That is, different electrode positions led to different ECG morphology. In this way, different signals were obtained in our study and different classification results were obtained and the results were compared.”*

**3-Comment of Reviewer2:** PPG Data Set

Can authors add the measurement units on the y-axis label in figure 1?

**Author’s Response**

We would like to thank to the reviewer for this valuable contribution. According to this comments we edited Figure 1 as follows in the “*PPG dataset*” section.

**4-Comment of Reviewer2:** Discussion

Even if future works aim to train and validate their 1D CNN model on a more balanced population in terms of sex and age, the authors should here discuss this crucial aspect regarding the clinical applicability of their work. As commented in the first revision, the population is sex-unbalanced (28 male vs 9 female). The incidence of atrial fibrillation is greater in males than in females, but this gap closes with advancing age (population in the present study aged between 51 and 90). Further sex-related differences in Photoplethysmography signals exist. At the present form, the proposed method is limited and it is not clinically applicable to a general population. Authors should deeply discuss these limitations in the discussion section.

**Author’s Response**

We would like to thank to the reviewer for this valuable contribution. According to this comment, we have revised the discussion section and added the following paragraphs.

*“In our current study, it was observed that the population was unbalanced in terms of sex (28 males, 9 females). In particular, it has been stated in various studies that thinner skin structure in women may affect PPG signals [41]. Neglecting this biometric factor may limit the performance of our model on women. The age range of the population used in our study (between 51-90) is also an important factor. The incidence of AF increases with age and age-related changes can be observed in PPG signals. Especially in elderly individuals, stiffening of peripheral vessels and changes in blood flow dynamics may affect the quality of PPG signals [41]. In addition, although the incidence of AF is higher in men, this difference decreases with increasing age and causes convergence between the sexes [42].*

*Despite these, advanced filtering techniques, calibration processes, adaptive algorithms and sensor improvements reduce these problems and increase PPG signal quality and reliability. Advanced deep learning algorithms have the ability to recognize and adapt to differences in sex and age-related data. This means that if the model is trained on a larger and more balanced population in the future, it can provide effective results for users of all ages and sexes. Indeed, the 17-layer CNN model used in our study can be optimized to better analyze sex and age-related differences. Validation of the model to be developed on more balanced populations will ensure that this application can be applied to a wider audience from a clinical perspective. In addition, the ability to monitor remotely and continuously with portable devices increases the potential of PPG signals in arrhythmia detection. Smartwatches can collect uninterrupted data during users' daily activities, and this data offers the advantage of providing continuous health monitoring without affecting the users' quality of life. In conclusion, the pro-posed 1D CNN model has significant potential to be integrated into the clinical workflow, but considering the population imbalance, sex and age-based biometric differences should be more represented in the model training. Future studies will include validation of the model on more balanced and larger populations, thus increasing its clinical applicability.”*

**5-Comment of Reviewer2:** In lines 552-553, authors stated “Signal quality can be affected by motion artifacts, electrode contact issues, and skin variability, making it difficult to obtain accurate PPG signals”. PPG is not acquired by electrodes, can authors correct their statement?

**Author’s Response**

We would like to thank to the reviewer for this valuable contribution. This sentence has been changed and combined with the paragraph mentioning the limitations of PPG, which was added in line with comment 4.

*“...However, the use of wearable devices also presents important limitations and challenges, such as signal quality and reliability. In addition to these limitations, factors such as the age and gender of people using wearable devices can also affect PPG signals.”*

**6-Comment of Reviewer2:** What do authors refer to with “invasive electrode placement (For Invasive EKG Devices)”? Non-invasive Electrocardiographic monitoring can be performed by dry electrodes and capacitive sensors for ECG, which can be incorporated in wearable and portable devices. The authors stated that ECG acquisition require contact with the subject, but the PPG

acquisition on the chest or on the wrist through smartwatches also require contact with

the device and possibly discomfort. Can Authors provide a more realistic and balanced comparison of wearable devices for ECG and PPG acquisition systems?

**Author’s Response**

According to this comments, a more proper comparison of ECG and PPG acquisition is presented in the following paragraph.

*“Achieving high accuracy in arrhythmia detection using PPG signals obtained from wearable devices offers numerous advantages; it supports remote monitoring, patient comfort, diagnosis with optical sensors, early detection of disease, and low cost-effectiveness. Moreover, real-time feedback increases the potential for long-term monitoring and wider access to health information. In contrast, the use of ECG signals comes with disadvantages such as complexity and hardware requirements, contact and discomfort, limited long-term monitoring, susceptibility to artifacts, and localization difficulties. ECG provides more accurate cardiological analysis by directly measuring the electrical activity of the heart, but even portable ECG devices have limited ease of use and are less practical for continuous monitoring. In contrast, PPG signals can be easily monitored with wearable devices at home, at work, or during sports, making PPG more accessible for continuous health monitoring and use in daily life. However, the use of wearable devices also presents important limitations and challenges such as signal quality and reliability. In addition to these limitations, factors such as age and sex of the individuals using wearable devices may also affect PPG signals.”*

**7-Comment of Reviewer2:** In table 4, authors should add ECG segment to the feature set of their work.

**Author’s Response**

We would like to thank to the reviewer for this valuable contribution. According to this comment, we added the following sentence to table 4.

*“ECG segment”*
